# Editorial: 40 years of relative age effects in sport: lessons from the past and directions for the future

**DOI:** 10.3389/fspor.2025.1745607

**Published:** 2025-12-04

**Authors:** Adam L. Kelly, Kathryn Johnston, Alexander B. T. McAuley, Joseph Baker

**Affiliations:** 1 Research for Athlete and Youth Sport Development (RAYSD) Lab, Birmingham City University, Birmingham, United Kingdom; 2 Tanenbaum Institute for Science in Sport, University of Toronto, Toronto, ON, Canada

**Keywords:** talent identification, talent development, athlete development, youth sport, RAE

## Introduction

2024 marked 40 years since Grondin, Deshaies, and Nault's (1) seminal work on relative age effects in sport. In their article, *Trimestres de Naissance et Participation au Hockey et au Volleyball* [Birth Trimesters and Participation in Hockey and Volleyball], they analysed the birthdate distribution of competitive youth and professional ice hockey and volleyball cohorts from North America. They revealed that players born in the early months of the selection year were overrepresented, whereas those born later in the year were underrepresented in ice hockey, although this pattern was not observed in their volleyball sample. Unaware of this article, Barnsley, Thompson, and Barnsley (2) shortly after reaffirmed these results in North American ice hockey, coining the term “relative age effects” (RAEs) to describe the phenomenon. They argued that the age group structures used to band athletes contributed to the loss of potentially talented individuals, as the abilities of relatively younger athletes were not accurately reflected in their performance due to age-related disadvantages. These foundational studies went on to inspire hundreds of relative age investigations worldwide.

Fast forward to today, and despite these early warnings, RAEs have proven remarkably persistent and difficult to mitigate in high-performance sport systems [see (3–6) for reviews; see (7–9) for editorials]. Notably, RAEs have been shown to be widespread across both boys' and girls' youth sport, significantly influencing the processes of identification, selection, and development of young athletes (10). These effects have, in turn, shaped long-term outcomes related to performance, participation, and personal development in sport (11, 12). Over the years, the relative age literature has reinforced several other key insights. For example, RAEs have tended to be more pronounced in popular sports such as basketball, soccer, ice hockey, and rugby (13), as well as in more competitive contexts as athletes progress into talent development systems (14).

The 40th anniversary of the original relative age studies offered a timely opportunity to reflect on developments in both research and practice. It also served as a prompt to look ahead and consider how athlete development systems can be improved to better serve all individuals, regardless of birth month. To help achieve this, we created this research topic as a platform for researchers and practitioners to reflect on RAEs and consider how we can move this area forward. As part of the call for articles, we felt it was critical for the field to adopt methodologies that not only reviewed the extensive body of literature across different sport settings, but also applied theoretical frameworks to deepen our understanding of how RAEs occur. Importantly, to influence practice beyond academic discussion, we believed it was essential to also capture studies that designed, implemented, and evaluated a range of relative age solutions across sporting environments. It was hoped this would encourage the development of targeted, evidence-informed interventions capable of addressing relative age disparities in meaningful, context-sensitive, and sustainable ways. Given that different sports are likely to require bespoke strategies [e.g., age- and size-based banding, birthday-banding, corrective adjustments, and competency-based banding for team, racket, timed, and combat sports, respectively (15)], further investigations into the mechanisms behind RAEs from multi-, inter-, and trans-disciplinary perspectives were also welcomed.

The purpose of this editorial is to summarise our research topic: *40 Years of Relative Age Effects in Sport: Lessons from the Past and Directions for the Future*. Based on the articles included, we have focussed on five key areas that emerged: (a) methodological reflections, (b) developmental pathways and career trajectories, (c) relative age and biological age, (d) relative age solutions, and (e) lessons from the past and directions for the future.

## Methodological reflections

In total, 85 authors from 14 countries contributed to the 23 articles included in this research topic. Studies were a mixture of quantitative (*n* = 16), qualitative (*n* = 2), and mixed method (*n* = 1) approaches. The majority of submissions were empirical studies (*n* = 20), with perspectives (*n* = 2; Grondin; Sweeney et al.) and a reflection (*n* = 1; Barnsley) also included. Of the empirical studies, 14 used analyses of large datasets to examine the prevalence of RAEs over at least one season, and of those articles, eight tracked RAEs over two seasons or more to examine historical trends. The remaining empirical studies used a mixture of measurement data (*n* = 2), questionnaire data (*n* = 1), interview data (*n* = 1), and some combinations of these approaches (*n* = 2). These empirical studies included a total of 213,789 participants (or individuals within the sample under investigation if secondary analysis was used), with 91,802 of those being women and girls, and 118,288 being men and boys. The individuals at the focus of the research came primarily from Europe and North America, with the two most represented countries being Germany (*n* = 4) and Spain (*n* = 3).

Within the articles, 16 focused on athletes and RAEs, two focused on the perceptions of interest holders on RAEs and potential solutions, one focused on the relative age of coaches, and one focused on the perspective of coaches. The most popular sport researched was, unsurprisingly, soccer (*n* = 11), followed by ice-hockey (*n* = 3), and handball (*n* = 2), while under researched sports such as fencing (Bonito et al.), orienteering (Ferriz-Valero et al.), squash (Kelly et al.), and swimming (Difernand et al.) were also examined. Women and girls were the sole focus of four studies, men and boys were the sole focus in eight studies, while another five studies included mixed genders, with the remaining two empirical studies not reporting the gender of participants.

With regard to procedures and analyses, some new and novel approaches were used. For example, Schorer et al. employed a retrospective and a longitudinal method. This approach helps to illuminate changes over time in birth quartile distribution, reiterating the need for relative age research to embrace longitudinal designs. It was also encouraging to see two studies (Kelly et al.; Smith et al.) employ interviews to gain the subjective experiences of those who influence and interact with RAEs. As well, one of the few studies that examined RAEs in the coaches themselves was included in this special issue (see Grondin et al.). This variety of samples and approaches presents a more nuanced and dynamic portrait of the impact of RAEs across multiple sport systems (see Table 1 for a summary of the articles included).

**Table 1:** Summary of the articles included in this research topic.

Author(s)	Submission Type	Method(s)	Number of Participants/Sample Under Observation	Role of Participant/Sample	Number of Men/Boys	Number of Women/Girls	Country of Participant/Sample	Sport	Level of Competition
Kelly et al.	Qualitative	Interviews	15	Coaches	14	1	England	Squash	Developing
Wang et al.	Quantitative	Large Dataset Analyses	10,485	Athletes	10,485	0	Mixed	Ice Hockey	Expert
Bonito et al.	Quantitative	Large Dataset Analyses	2,791	Athletes	1,575	1,216	Mixed	Fencing	Mixed
Smith et al.	Qualitative	Interviews and Questionnaire	15	Athletes	0	15	Canada	Soccer	Developing
Wenger and Csapo	Quantitative	Measurement	98	Athletes	98	0	Austria	Soccer	Developing
Lemoyne et al.	Quantitative	Large Dataset Analyses	4,306	Athletes	4,306	0	Mixed	Ice Hockey	Mixed
Thieschäfer et al.	Quantitative	Large Dataset Analyses	4,599	Athletes	2,340	2,259	Germany	Handball	Developing
Kelly et al.	Mixed Method	Delphi	15	Researchers and Practitioners	Not Reported	Not Reported	Mixed	Soccer	N/A
Ferriz-Valero et al.	Quantitative	Large Dataset Analyses	7,731	Athletes	4,318	3,109	Spain	Orienteering	Mixed
Morganti et al.	Quantitative	Large Dataset Analyses	1,565	Athletes	1,565	0	Mixed	Soccer	Developing
Pérez-González et al.	Quantitative	Large Dataset Analyses	1,634	Athletes	0	1,634	Mixed	Soccer	Expert
Kelly et al.	Qualitative	Questionnaire	134	Interest Holders	Not Reported	Not Reported	Mixed	Soccer	N/A
Schorer et al.	Quantitative	Large Dataset Analyses	1,015	Athletes	542	473	Germany	Handball	Mixed
Peña-González et al.	Quantitative	Large Dataset Analyses	1,120	Athletes	1,120	0	Spain	Soccer	Developing
Barnsley	Opinion	N/A	N/A	N/A	N/A	N/A	N/A	N/A	N/A
Difernand et al.	Quantitative	Large Dataset Analyses	160,861	Athletes	79,144	81,717	France	Swimming	Mixed
Grondin et al.	Quantitative	Large Dataset Analyses	3,380	Coaches	Not Reported	Not Reported	Canada	Ice Hockey	Mixed
Peñín-Grandes et al.	Quantitative	Measurement	80	Athletes	80	0	Spain	Soccer	Expert
Sweeney et al.	Perspective	N/A	N/A	N/A	N/A	N/A	N/A	N/A	N/A
Smith et al.	Quantitative	Large Dataset Analyses	1,378	Athletes	0	1,378	Germany	Soccer	Developing
Grondin	Perspective	N/A	N/A	N/A	N/A	N/A	N/A	N/A	N/A
Heilmann et al.	Quantitative	Large Dataset Analyses	3,174	Athletes	3,174	0	Germany	Soccer	Developing
Brustio et al.	Quantitative	Large Dataset Analyses	9,527	Athletes	9,527	0	Mixed	Soccer	Mixed

## Developmental pathways and career trajectories

As expected, there were several studies that examined RAEs across athlete development pathways, particularly in soccer. While some of these used typical approaches to identifying and considering RAEs (e.g., Heilmann et al.; Peña-González et al.), the samples and research questions explored extended our knowledge to new populations. For instance, Pérez-González et al. showed RAEs existed in four of the five top five European women's football leagues, highlighting the knock-on effects of relative age at senior levels. Other studies used untraditional approaches to examine the impact and development of RAEs, such as the use of survival analysis to examine sport dropout in French swimmers by Difernand et al.

Perhaps most notably were the impressive number of studies examining RAEs from longitudinal and developmental perspectives. Many of these (e.g., Brustio et al.; Lemoyne et al.; Morganti et al.; Schorer et al.) emphasised the need to use longitudinal designs to track changes in athlete populations over the extensive time course of athlete development. As an example, Wang et al. used cohort data over 44 years to assess how time impacts RAEs, helping to better understand the career trajectories of professional ice hockey players. Relatedly, Smith et al. emphasised that RAEs can take multiple forms over a long period, reflecting the reality that what appears to be a very simple and straightforward phenomenon is, in fact, multifaceted and complex with varied influences and implications.

## Relative age and biological age

Several papers analysed the role of relative age and biological age. This work emphasised that, while they are both vital to consider during the identification, selection, and development processes in sport (16), they are two separate constructs that operate independently (17). Out of three studies, two focused on soccer, with one comprised of participants in a Spanish professional academy (Peñín-Grandes et al.), and the other the first examination of relative and biological age constructs in an Austrian soccer context (Wenger and Csapo). The third study investigated the association of maturation and relative age with talent selection in German youth handball players, whereby the authors presented evidence that a unique inter-play may exist between the constructs, as it could be crucial for relatively younger players to mature earlier to increase their selection odds (Thieschäfer et al.).

Research on a range of sports including cricket (18), handball (19), rugby (20), and soccer (21) has showed “bio-banding” [i.e., a format that groups athletes based on maturity status rather than chronological age (22)] to be an effective way at mitigating biological age effects. It is important to emphasise, though, that bio-banding is a potential solution for maturation-related biases, and not for RAEs. Thus, integrating bio-banding alongside relative age solutions, or combining solutions together [e.g., (23–26)], may be fruitful areas for future research.

## Relative age solutions

A persistent call from relative age researchers, including in this research topic, is identifying potential solutions. Although many authors reiterated these sentiments in their articles, only three empirically explored this area, with only one evaluating the implementation of the solution. Building on previous quantitative work on “birthday-banding” (i.e., each individual athlete competes with those of the same chronological age, moving up to the next birthday-band on their birthday), which corresponded with no RAEs exist in the England Squash Talent Pathway (27), Kelly et al. explored coaches' perspectives of the approach. They noted how birthday-banding produced fairness for athletes who might have been removed due to their birthday, offering a possible relative age solution for other racket sports.

The Royal Netherlands Football Association (KNVB) relative age solutions project also contributed two studies. First, following a call to action for interest holders to propose relative age solutions to KNVB, almost 200 proposals were categorised into 13 independent approaches (Kelly et al.). Interestingly, whilst no new suggestions outside the existing literature were proposed, only two have been empirically tested in soccer (24, 28). Second, Kelly et al. evaluated these 13 proposed solutions, using a two-round adapted e-Delphi study with 15 international experts (i.e., researchers and practitioners). Generally, highly rated solutions perceived to effectively moderate RAEs were expected to be more challenging to implement, whereas those more feasible to implement were considered less effective. Results also showed regular disagreement amongst the international experts, highlighting that achieving consensus on possible relative age solutions may be challenging.

## Lessons from the past and directions for the future

As part of this research topic, we were fortunate to include four decades worth of reflections from the inaugural researchers of RAEs—Professor Simon Grondin and Professor Roger Barnsley. In his opinion article, Grondin closed by repeating a key takeaway message from his original 1984 paper, emphasising how even after 40 years, many sport systems have still not comprehended this simple message: “It must be remembered that two children born in the same year do not necessarily have the same age” (1). Barnsley repeated these sentiments in his perspective article, showcasing that the data they published from the 1983 Western (WHL) and Ontario (OHL) Major Junior A Hockey Leagues (2) and from the 1989 FIFA U17 and U20 World Cups (29) remains almost identical, whereby those born in the first three months are overrepresented (cumulative ∼40%) and those born in the last three months of the selection year are underrepresented (cumulative ∼12.5%).

Overall, it is fair to say that whilst our understanding of RAEs has grown considerably over the last four decades, not a lot has changed in practice. Indeed, both Grondin and Barnsley expressed their disappointment with how the applied field has not utilised the comprehensive research knowledge. We call for researchers and practitioners to work closer together to be solution-focused, helping create more developmentally appropriate learning environments for all young athletes to achieve their potential in sport, irrelevant of their birthdate.

## Summary

Although a large body of research has documented the presence of RAEs across various sporting contexts, and despite the ongoing recommendations from researchers and practitioners, efforts to design, implement, and evaluate effective and feasible solutions have been limited. Moreover, while several theoretical explanations have been proposed and greater clarity provided on the differences between biological and relative age, there remains a lack of empirical studies that clearly identify the underlying causes of RAEs in sport. Such studies would be invaluable in guiding the development of context-sensitive interventions (9). In addition to the continuously growing number of relative age studies across a variety of sport environments, an important next step will be to systematically review the literature, using meta-analyses, in widely researched sports. This will aid the development of sport-specific consensus on RAEs and outline next steps.

Finally, we call for researchers and practitioners to focus their efforts not only on replicating relative age research in different contexts, but also ensuring each study meaningfully adds new knowledge to the field. Researchers should use relative age studies as an opportunity to examine multi-, inter-, and trans-disciplinary features that contribute towards RAEs, as well as explore the direct and indirect effects of relative age to help explain how they occur. Doing so will inform strategies that are effective and feasible for sport-specific contexts, helping to create youth sport environments that foster long-term performance, participation, and personal development outcomes for every young person, regardless of when they are born.

It was a pleasure to assemble this research topic. We hope the reader finds the articles useful in helping to advance their own research and practice. Thanks to the authors, reviewers, and participants for their support—without their efforts, advancing our knowledge of RAEs would not have been possible.
